# Monitoring and mitigation of toxic heavy metals and arsenic accumulation in food crops: A case study of an urban community garden

**DOI:** 10.1002/pld3.198

**Published:** 2020-01-14

**Authors:** Andrew M. Cooper, Didra Felix, Fatima Alcantara, Ilya Zaslavsky, Amy Work, Paul L. Watson, Keith Pezzoli, Qi Yu, Dan Zhu, Alexander J. Scavo, Yasman Zarabi, Julian I. Schroeder

**Affiliations:** ^1^ Division of Biological Sciences, Cell and Developmental Biology Section University of California, San Diego La Jolla CA USA; ^2^ Spatial Information Systems Laboratory San Diego Supercomputer Center La Jolla CA USA; ^3^ UC San Diego Library San Diego Supercomputer Center La Jolla CA USA; ^4^ Global Action Research Center San Diego CA USA; ^5^ Department of Urban Studies and Planning Bioregional Center for Sustainability Science, Planning and Design University of California, San Diego La Jolla CA USA; ^6^ Hubei Key Laboratory of Genetic Regulation and Integrative Biology School of Life Sciences Central China Normal University Wuhan China

**Keywords:** arsenic, cadmium, heavy metal, ion transport, ionomics, Pb lead, phytoremediation, tomato/Solanum lycopersicum

## Abstract

Urban community gardens have increased in prevalence as a means to generate fresh fruits and vegetables, including in areas lacking access to healthy food options. However, urban soils may have high levels of toxic heavy metals, including lead and cadmium and the metalloid arsenic, which can lead to severe health risks. In this study, fruit and vegetable samples grown at an urban community garden in southeastern San Diego, the Ocean View Growing Grounds, were sampled repeatedly over a four‐year time period in order to measure potential contamination of toxic heavy metals and metalloids and to develop solutions for this problem. Metal nutrient, heavy metal, and metalloid concentrations were monitored in the leaf and fruit tissues of fruit trees over the sampling period. Several of the fruit trees showed uptake of lead in the leaf samples, with Black Mission fig measuring 0.843–1.531 mg/kg dry weight and Mexican Lime measuring 1.103–1.522 mg/kg dry weight over the sampling period. Vegetables that were grown directly in the ground at this community garden and surrounding areas showed arsenic, 0.80 + 0.073 mg/kg dry weight for Swiss chard, and lead, 0.84 ± 0.404 mg/kg dry weight for strawberries, in their edible tissues. The subsequent introduction of raised beds with uncontaminated soil is described, which eliminated any detectable heavy metal or metalloid contamination in these crops during the monitoring period. Recommendations for facilitating the monitoring of edible tissues and for reducing risk are discussed, including introduction of raised beds and collaborations with local universities and research groups.

## 1 INTRODUCTION

In recent years, there has been a major resurgence in the prevalence of urban community gardens (Alaimo, Packnett, Miles, & Kruger, 2008; McCormack, Laska, Larson, & Story, 2010; Preer, Sekhon, Stephens, & Collins, 1980). Urban gardening has increased even more rapidly worldwide over the last 20 years (Mitchell et al., 2014). Motivations for gardening can vary greatly but often include a desire for lower cost, and higher quality fruits and vegetables (Sterrett, Chaney, Gifford, & Mielke, 1996). The need for access to healthy food sources through community gardens is greater in areas classified as food deserts. A food desert is defined by the USDA as an urban community or neighborhood that is distant from access to affordable foods for a full healthy diet (USDA, 2009). Food deserts are most often located in regions with lower socioeconomic status and are comprised of underserved and underrepresented communities (Ferdinand & Mahata, 2017; Smith, 2016). Members of these communities have limited resources and access to transportation, making urban gardening one of the main options available for access to healthy food and vegetables. There are a number of different theories about the development and increase in food deserts in urban communities in the United States, including rapid shifts in inner‐city demographics beginning in the 1970s, large‐chain supermarkets making smaller inner‐city stores non‐viable, and inaccurate perceptions about possible financial gain, land access, and safety (Nyden, Lukehart, Maly, & Peterman, 1998; Walker, Keane, & Burke, 2010).

All of these theories likely influence part of the process, as affluent households have left the inner cities to establish higher income neighborhoods large‐chain stores moved into the outskirts of these areas (Guy, Clarke, & Eyre, 2004; Wienk, 1979). The combination of lowered median income in the inner cities and competition with large‐chain stores that can offer wider options at lower prices drives smaller, independently owned stores out of business. Combining these factors with the lack of personal vehicles and problematic transportation in the United States makes access to these chain supermarkets on the outskirts of city neighborhoods difficult (Curtis & McClellan, 1995). Studies have shown that with decreased median income, access to healthy food options and supermarkets decreases, and this difference becomes even more dramatic when racial and ethnic makeup of the neighborhood is taken into account (Chung & Myers, 1999; Morland, Wing, Roux, & Poole, 2002; Powell, Slater, Mirtcheva, Bao, & Chaloupka, 2007). Given the increasing difficulty and cost associated with obtaining healthy food options, people are increasingly moving toward the idea of community gardening as a means to fill this need.

While there are many benefits to urban gardening, there are also associated risks (Preer, Akintoye, & Martin, 1984). Urban gardens are often established at sites known as brownfields. Brownfields are defined by the Environmental Protection Agency (EPA) as property whose use is complicated by the presence or potential presence of a hazardous substance or contaminant (EPA, 2017). EPA estimates show that there are more than 450,000 brownfields in the United States, many in urban areas. These sites are attractive for conversion to community gardens as they are often the only unutilized land in the area (De Sousa, 2003; Defoe, Hettiarachchi, Benedict, & Martin, 2014). However, the presence of toxic heavy metal and metalloid contaminants, such as arsenic, lead, and cadmium, at these sites complicates their use for growing food products (Hough et al., 2004; Mielke et al., 1983).

Heavy metal and arsenic contamination can come from diverse sources depending on location and historic land use (Alloway, 2013; Harrison, Laxen, & Wilson, 1981). Agricultural land often has increased levels of arsenic, lead, and cadmium due to application of fertilizers and now outlawed pesticides. In urban areas, major sources of lead contamination include lead‐based paints, automotive emissions, and local industries such as smelters and manufacturing (Clarke, Jenerette, & Bain, 2015; Thornton, 2009). In addition, soil contamination can be increased due to contaminated water running through urban areas from other regions.

Heavy metals and arsenic can enter the human body through defined avenues, including inhalation of contaminated dust, direct ingestion of contaminated soil on the surface of foods, and ingestion of food plants containing heavy metals or arsenic due to contamination of the growth site. In areas with no contamination of drinking water, it has been proposed that ingestion is the highest risk of arsenic exposure with vegetables having been reported to make up the largest percentage of exposure, followed by fruit and fruit juices, and rice (Chain EPoCitF, 2009; Xue, Zartarian, Wang, Liu, & Georgopoulos, 2010). Long‐term exposure to arsenic, lead, and cadmium can result in a wide range of detrimental health effects including hypertension, diminished lung function, increased risk of liver disease, diverse cancers, and developmental defects after exposure in children and pregnant women (Alissa & Ferns, 2011; Cave et al., 2010; Ghatak et al., 2011; Heck et al., 2009; Huang et al., 2013; Hyder et al., 2013; Ohta, Ichikawa, & Seki, 2002; Satarug, Garrett, Sens, & Sens, 2011; Sherief et al., 2015). Due to these severe health risks, the development of urban community gardens should be undertaken with careful planning in order to minimize dangers associated with heavy metal and arsenic contamination. In addition, initial testing of heavy metal contamination in the soil, as well as ongoing monitoring of plant tissue contamination, would be ideal.

**Figure 1:**
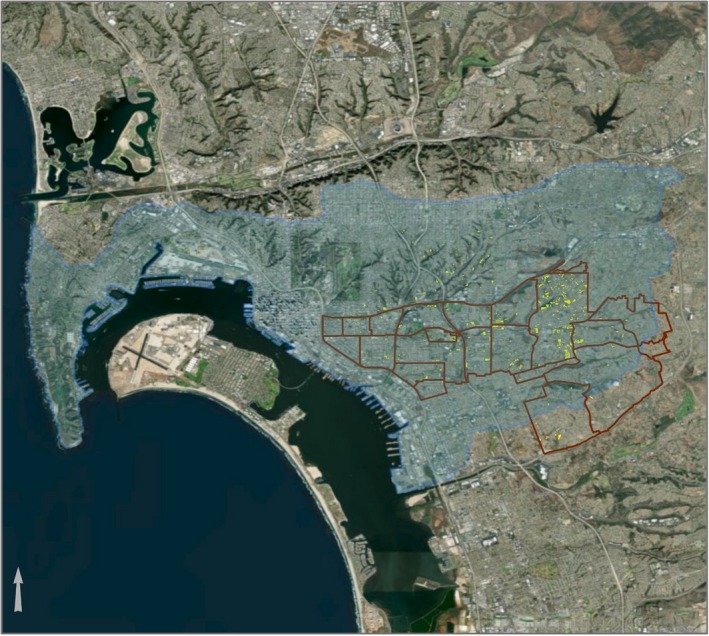
Map of Southeastern San Diego. A map of Southeastern San Diego with vacant lots indicated by yellow polygons. The arrow at the bottom left indicates north direction

There are over 810 vacant lots in southeastern San Diego and surrounding areas, including City Heights, Golden Hill, and Mid‐City Eastern (Figure 1). Many of these sites are located within food deserts and could be potential sites for urban gardens. In this study, one specific brownfield site in southeastern San Diego, the Ocean View Growing Grounds (OVGG), has been developed as an urban community garden and greenspace. From 1953 until at least 1964, the property was developed as a nursery; however from 1980 until 2012, the site was vacant but was used as additional parking/storage for the automotive repair facility immediately adjacent to the west of the site, which may have introduced a variety of contaminants into the soil. This site has been developed through a collaboration between the community and the Global Action Resource Center (ARC) to provide access to healthy fruits and vegetables, as well as a location for community events learning. The UC San Diego Superfund Research Program, in partnership with the Community Engagement and Research Translation Cores, has developed an edible plant tissue testing program to monitor and analyze the heavy metal and arsenic uptake in food plants being grown at the Ocean View Growing Grounds. This information can be used to better inform and design the growth conditions at the site.

## 2 METHODS

### 2.1 Plant sample collection and testing

Plant tissue samples were collected from plants growing at the Ocean View Growing Grounds in partnership with UC San Diego's Bioregional Center for Sustainability Science, Planning and Design, with public access. Leaf and fruit tissues were sampled from fruit trees repeatedly over a 4‐year period to monitor heavy metal and arsenic uptake. The blood orange tree had leaf samples collected at five different times, and fruit samples were collected as described. The Mexican lime had leaf tissue collected at six different times and fruit samples collected as described. The Black Mission fig had leaf tissue collected at six different times, and fruit samples were collected at three different times. For leaf samples, three separate samples of tissue were collected at each collection time point, with each sample consisting of multiple leaves randomly gathered from trees. For fruit samples, three individual fruits were collected and separately tested. Seasonal ground grown crops (Figures 4 and 5) consisted of a single sampling during the growing season, with three separate tissue samples collected and tested. For each collection, the three separately collected and tested samples were averaged and the standard deviation was calculated. After collection, the tissue samples were rinsed with double‐distilled water then wrapped in unused clean paper sheets and dried at 60ºC (140ºF) for 5 days, then digested in 68% nitric acid. Fresh weights prior to drying of samples and dry weights after drying were determined for the reported tissue and place species. The digested tissue samples were then diluted 1:20 with double‐distilled water. The heavy metal content of the diluted samples was determined with a Perkin Elmer Optima 3000 DV ICP‐OES at the Scripps Institution of Oceanography Inductively Coupled Plasma Optical Emission Spectrophotometer Facility. Claritas PPT Multi‐Element Solution 2 (Cat#: CLMS‐2) was used as a measurement standard for heavy metal and arsenic quantification. The detection limit for As and Pb was ~0.2 mg/kg.

### 2.2 Soil sample collection and testing

**Figure 2:**
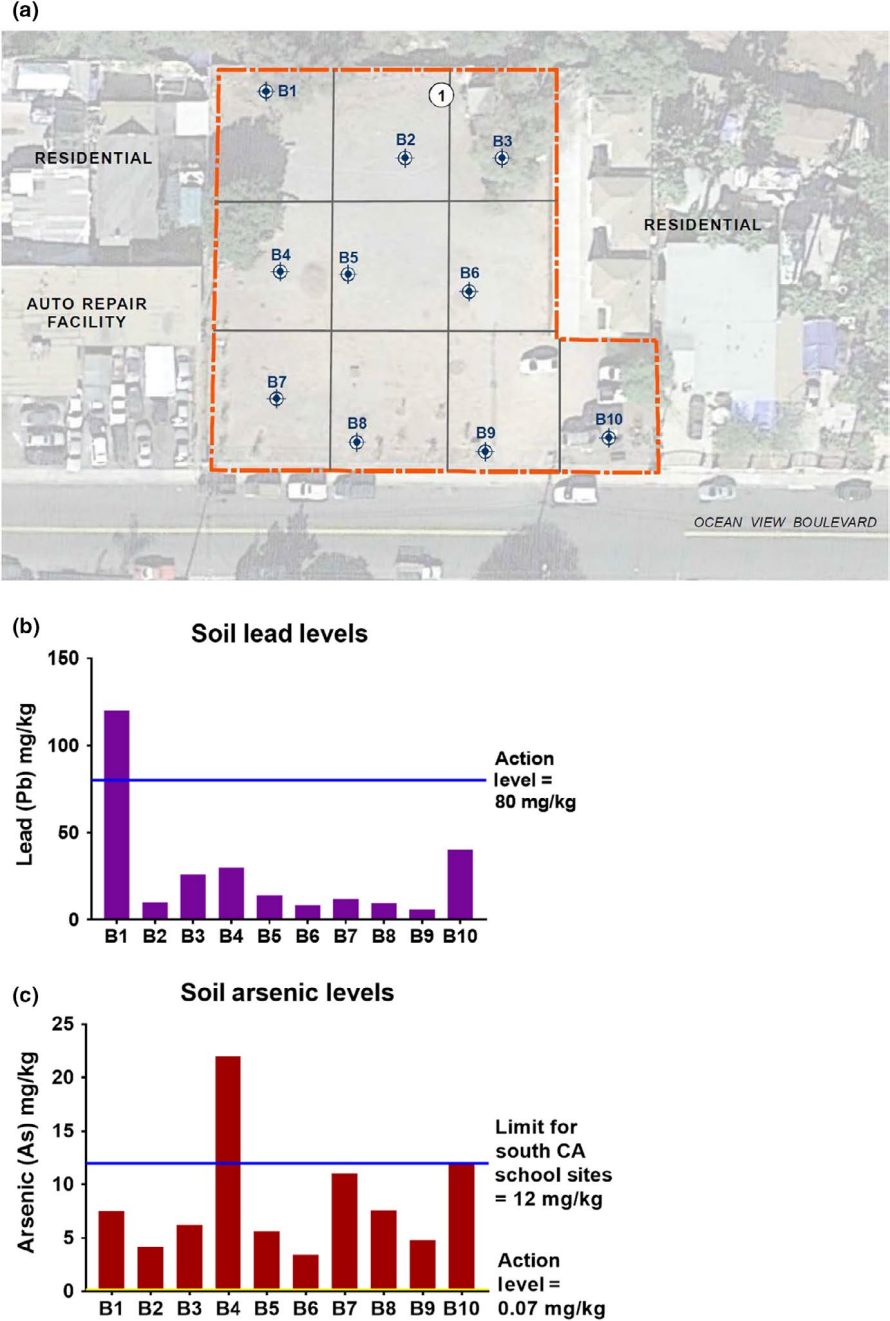
Location for ten soil samples and lead/arsenic levels in soil. (a) Soil samples taken from 10 locations throughout the Ocean View Grounding Grounds were measured for heavy metal and arsenic content. (b) Lead and (c) arsenic levels are shown from all 10 sampled locations. The action levels of 80 mg/kg dry weight for lead and 0.07 mg/kg dry weight for arsenic are indicated in each graph

Soil samples were collected and measured by Wallace and Ninyo & Moore Geotechnical and Environmental Sciences Consultants at the beginning of the project by 2014. Soil testing was done once near the beginning of the project using funds obtained from a USEPA assessment area‐wide grant. Each soil sample was collected from 0.5 ft below ground surface using a hand auger that was decontaminated between each collection. Hand auger boring locations were selected using a grid system, and then, the locations were modified within each grid based on the current and planned configuration of the community garden. Boring locations are depicted in Figure 2 (Data S1). In between each sample, all equipment was decontaminated using a three‐step process: (a) non‐phosphate detergent in tap water wash, with brush, (b) tap water rinse, and (c) distilled water rinse. All decontamination was performed in a pre‐designated area on plastic sheeting. Soil samples were stored in laboratory‐supplied 4‐ounce glass jars and transported on ice to Orange Coast Analytical, Inc. Quantification of the heavy metal and metalloid content was determined at Orange Coast Analytical, Inc. using the USEPA method 6010B (USEPA, 1997).

### 2.3 Figure 1 map

The map of southeastern San Diego in Figure 1 was generated using ArcGIS^®^ software by Esri. For more information about Esri^®^ software, please visit http://www.esri.com. The map shows the following information layers:

- Vacant and Undeveloped Land. Source: “Land_Use_2015”, SanGIS Data Warehouse. 2014–10–02. San Diego Geographic Information Source. Downloaded, July 12, 2019, http://www.sangis.org/download/index.html
- San Diego Neighborhood Boundaries. Source: the following resources: SanGIS. "Neighborhoods_SD", SanGIS Data Warehouse. June 1, 2012. San Diego Geographic Information Source ‐ JPA. Downloaded, July 12, 2012, http://www.sangis.org/download/index.html.
- Watersheds. Source: U.S. Geologic Survey (USGS), SanGIS. "Watersheds" SanGIS Data Warehouse. June 1, 2016. San Diego Geographic Information Source ‐ JPA. Downloaded, July 1, 2019, http://www.sangis.org/download/index.html.
- Background imagery. Sources: World Imagery Sources: Esri, DigitalGlobe, GeoEye, i‐cubed, USDA FSA, USGS, AEX, Getmapping, Aerogrid, IGN, IGP, Swisstopo, and the GIS User Community.

## 3 RESULTS

### 3.1 Soil sample testing

Soil contamination levels at the Ocean View Growing Grounds were analyzed, including heavy metals and metalloids. Figure 2a shows the collection locations for the ten soil samples. B1 was the only soil sample that showed lead levels above the California Human Health Screening Levels (CHHSLs) action level of 80 mg/kg (CalEPA, 2010), with lead levels of 120 mg/kg (Figure 2b). The general background level of lead contamination in California is 29.7 ± 1.3 mg/kg (Smith et al., 2013). All samples collected showed arsenic levels above the CHHSLs action level of 0.07 mg/kg (CalEPA, 2010), ranging from 3.4 to 22 mg/kg (Figure 2c). The general background levels of arsenic contamination in Southern California, without anthropogenic input, are between 0.6 and 11 mg/kg, and the safety limit for school sites in Southern California is 12 mg/kg (Bradford et al., 1996) (Figure 2c).

The soil pH ranged from 6.02 to 7.77 with an average of 6.87 (see Data S1). The salinity of the soil was moderate, ranging from 0.19 to 4.79 millimho/cm with an average of 1.36 millimho/com. There was relatively high chloride at around 502 parts per million. A surface composite sample analyzed showed high fertility except for low mineral nitrogen (see Data S1).

### 3.2 Measurement of heavy metals in fruit trees

**Figure 3:**
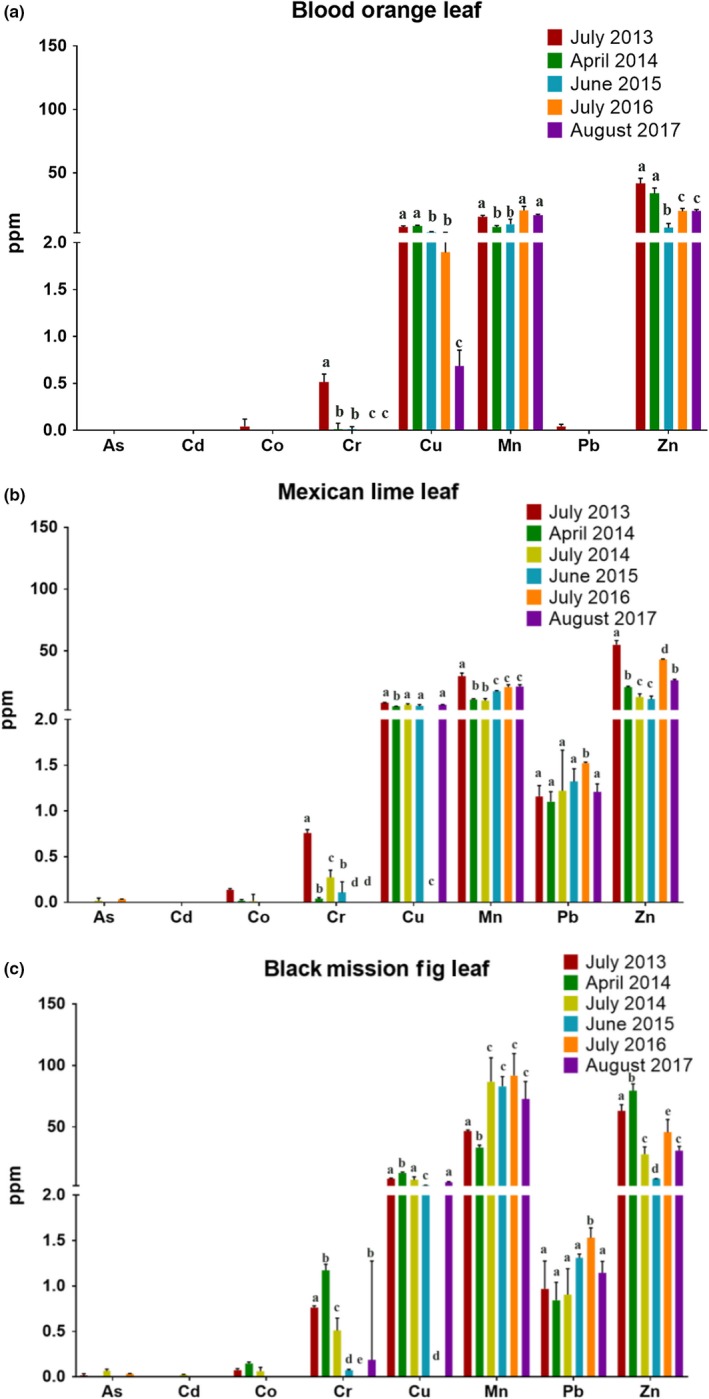
Metal and Arsenic content of leaf samples taken from Ocean View Growing Grounds. Metal and arsenic content of leaf samples taken from the indicated fruit tree species grown at the Ocean View Growing Grounds. (a) Blood Orange Leaf (b) Mexican Lime Leaf (c) Black Mission Fig Leaf (As: arsenic, Cd: cadmium, Co: cobalt, Cr: chromium, Cu: copper, Mn: manganese, Pb: lead, Zn: zinc). Each bar indicates the mean ± *SD* of three samples collected on the indicated dates (ppm = mg/kg dry weight). Letters on the top of bars show grouping from one‐way ANOVA analyses, analyzed for each element separately, for elements in which any sample was above the detection limit

Within the Ocean View Growing Grounds, there are two areas composed of fruit trees, called Food Forest #1 and Food Forest #2. Repeated samples of leaf tissue have been taken from these trees starting shortly after their planting, with three to six collections over four years. Figure 3 shows repeated sampling from three tree species within Food Forest #1, which is the older of the two food forests. A variety of fruit trees sampled, including the blood orange shown in Figure 3a, had no detectable accumulation of arsenic (As), lead (Pb), and cadmium (Cd) in leaves, but consistently detectable levels of non‐toxic metals, including copper (Cu), manganese (Mn), and zinc (Zn). In a subset of the fruit trees present, we did find detectable accumulation of lead from the soil in the leaves, including Mexican lime (Figure 3b) and Black Mission fig (Figure 3c) trees. The detected lead levels in the Black Mission fig leaves and Mexican lime leaves have been relatively stable over the six collected samples spanning four years (Figure 3b,c).

**Figure 4:**
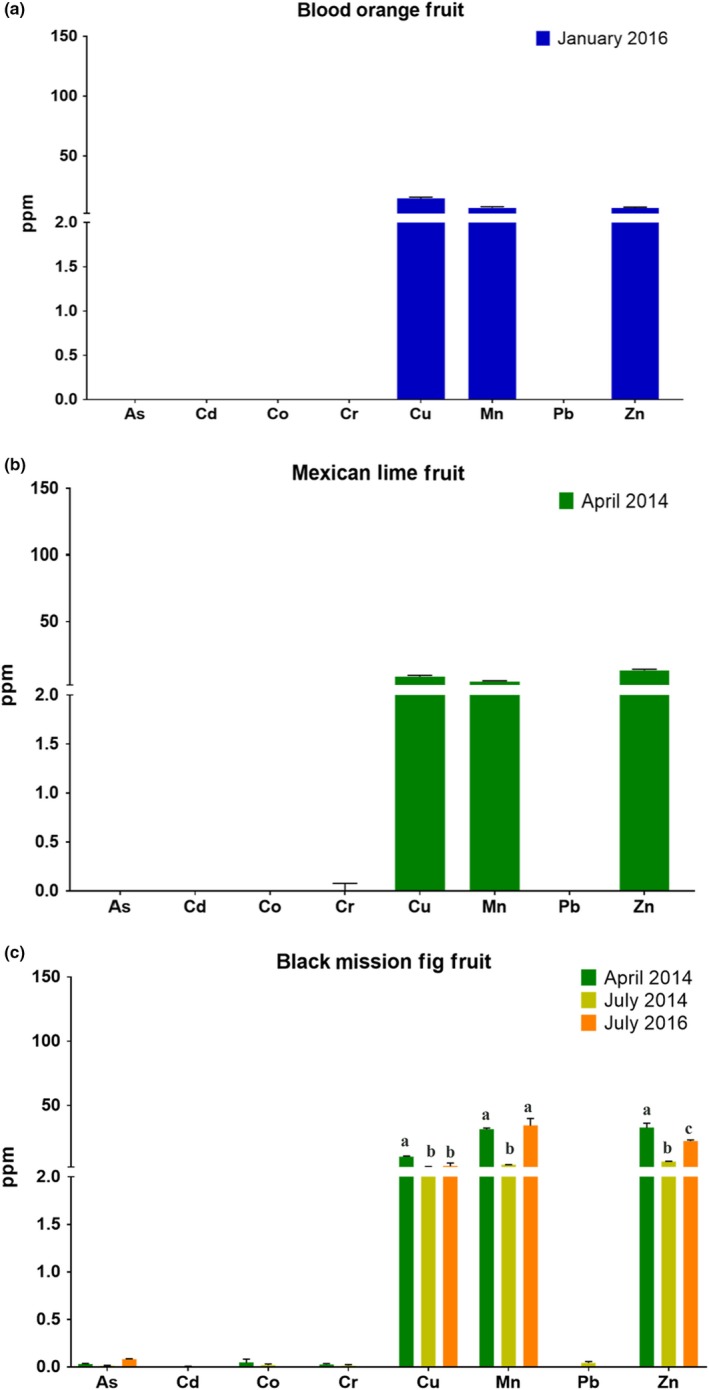
Metal and arsenic content of fruit samples taken from Ocean View Growing Grounds. Metal and arsenic content of fruit samples taken from the indicated fruit trees grown at the Ocean View Growing Grounds. (a) Blood Orange Leaf, (b) Mexican Lime Leaf, (c) Black Mission Fig Leaf. (As: arsenic, Cd: cadmium, Co: cobalt, Cr: chromium, Cu: copper, Mn: manganese, Pb: lead, Zn: zinc). Each bar indicates the mean ± *SD* of three samples collected on the indicated dates. (ppm = mg/kg). Letters on the top of bars in (c) show grouping from one‐way ANOVA analyses, analyzed for each element separately, for elements in which any sample was above the detection limit

Fruit samples were collected from these same trees within the Food Forests. There were limited samples of edible tissues due to low fruit yield. Heavy metal measurements from the edible fruit samples were collected and showed no detectable toxic heavy metal or arsenic contamination (Figure 4). The lack of detectable heavy metals and arsenic in the fruit samples was found regardless of whether the leaf tissue of the trees showed contamination (Mexican lime, Figure 4b and Black Mission fig, Figure 4c), or showed no contamination (blood orange, Figure 4a). Note that heavy metals and arsenic are known to accumulate in fruits, and therefore, measurements of fruits are important, as this may depend on the concentration of toxicants in leaves and soils, soil chemistry, age of trees, and diverse other parameters (see: Discussion). The fruits from all of these trees showed accumulation of nutrient metals (Zinc: Zn, Cooper: Cu, Manganese: Mn).

### 3.3 Measurement of heavy metals in seasonal crops

**Figure 5:**
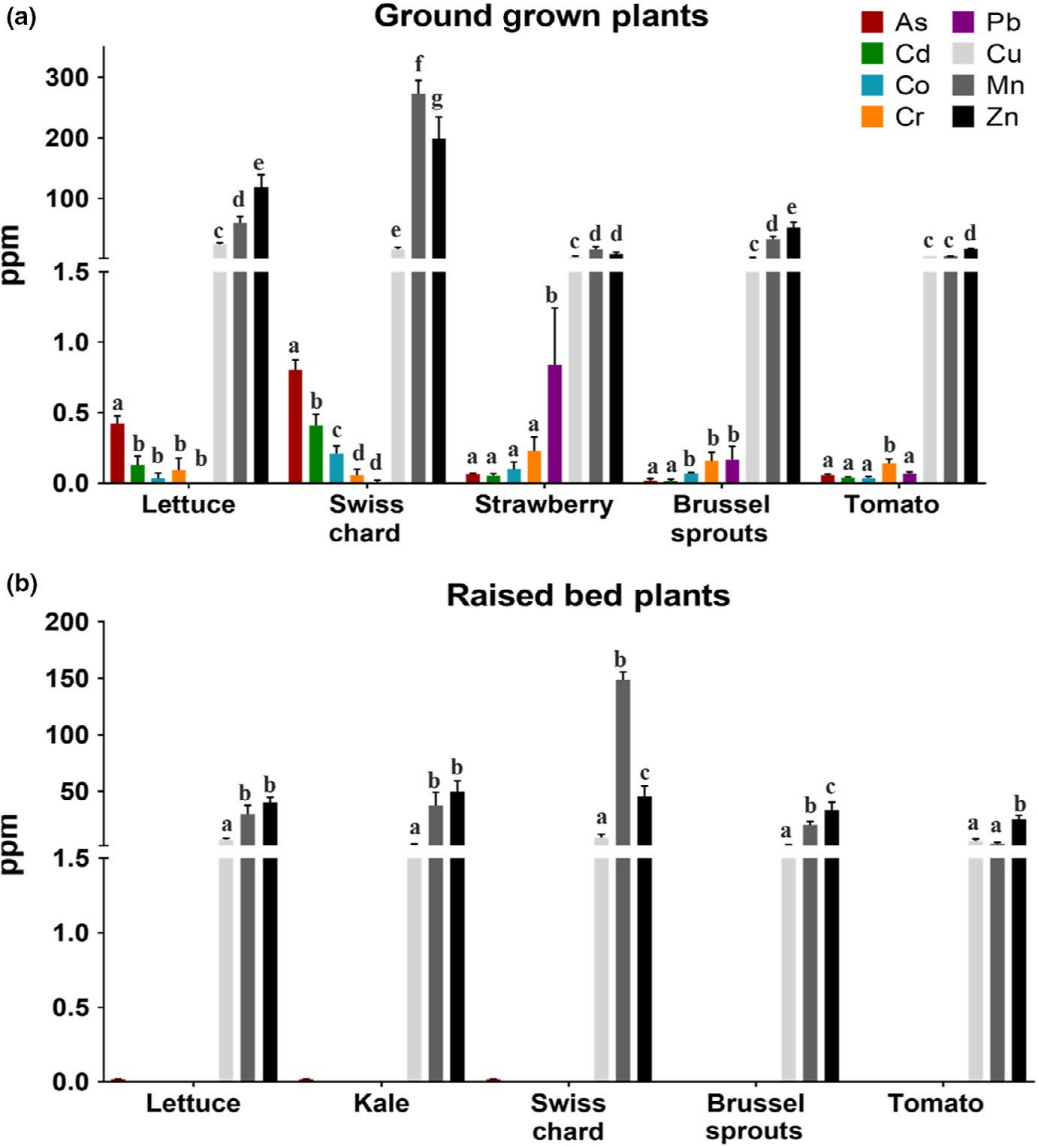
Metal and arsenic content of edible samples taken from Ocean View Growing Grounds. Metal and arsenic content of edible tissue samples taken from the indicated plants growing at the Ocean View Growing Grounds, grown either (a) directly in the soil or (b) in raised beds. The nutrients copper (Cu), manganese (Mn), and zinc (Zn) are indicated in gray tones. Toxic metals and arsenic are indicated in color (As: arsenic, Cd: cadmium, Co: cobalt, Cr: chromium, Pb: lead). Each bar indicates the mean ± *SD* of three samples (ppm = mg/kg dry weight). Letters on the top of bars show grouping from one‐way ANOVA analyses, analyzed for each element separately, for elements in which any sample was close to or above the detection limit. In (b), sub‐threshold heavy metal and arsenic traces were not clearly detected for plants grown in raised beds, and therefore, no ANOVA groupings are shown, despite attempts to identify these toxicants in these plant samples

In addition to the fruit trees present within the two food forests, vegetables have been planted at the Ocean View Growing Grounds and nearby backyard gardens. Figure 5a shows metal levels in a variety of edible plants grown directly in the community garden soil. In leafy green vegetables, including Swiss chard and lettuce, detectable levels of arsenic (As) were found while strawberries showed detectable levels of lead (Pb). Specifically, the Swiss chard showed 0.80 ± 0.073 mg/kg dry weight arsenic, while the lettuce showed 0.42 ± 0.055 mg/kg dry weight arsenic. Strawberries showed 0.84 ± 0.404 mg/kg dry weight lead. Other vegetables including Brussel sprouts and tomato showed no detectable level of heavy metals and metalloids at or below thresholds.

**Figure 6:**
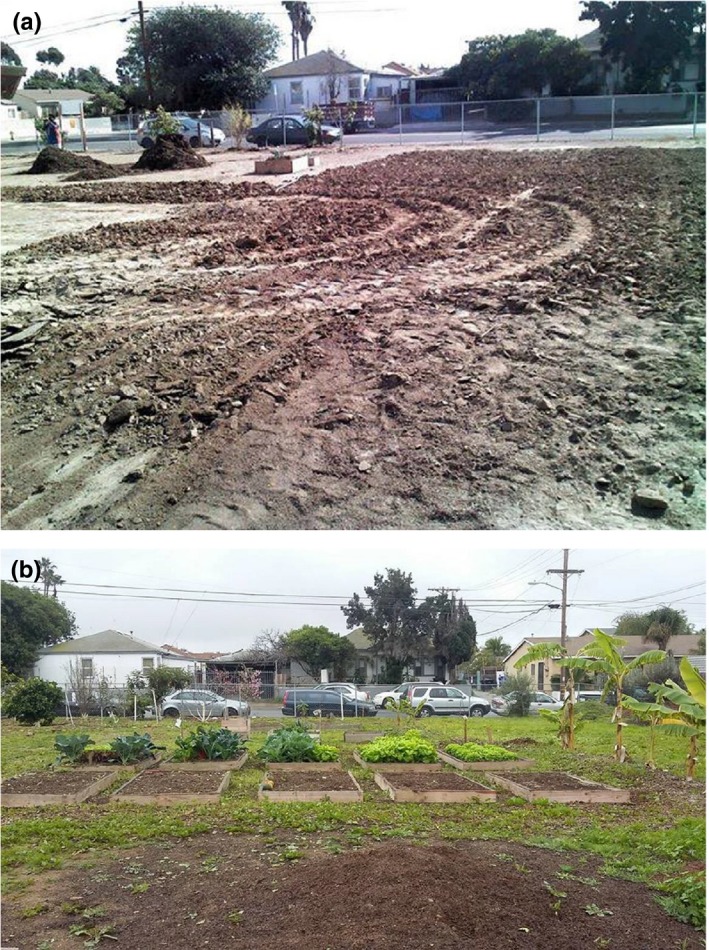
Ocean View Growing Ground before and after raised beds. Ocean View Growing Grounds before and after the introduction of raised beds. (a) No raised beds and (b) raised beds

In an effort to minimize risks associated with ingesting vegetables grown directly in the heavy metal contaminated ground at the Ocean View Growing Grounds, raised beds were constructed in the Fall of 2014 using certified organic, toxicant free‐soil from City Farmers (https://www.cityfarmersnursery.com/soil) (Figure 6). To minimize transfer of contaminants between existing soil and that brought in for the raised beds, the top layer of soil at the site was scraped away. Due to limitation of available resources, no boundary layer was placed between the raised bed soil and existing site, reinforcing the need for continued monitoring (see Discussion). All seasonal fruits and vegetables are now grown in these raised beds. Heavy metal and arsenic measurement of vegetables grown in the raised beds, including leafy green vegetables such as lettuce, kale, and Swiss chard (Figure 5b), showed no detectable levels of heavy metals or arsenic. In contrast, nutrient metals were detected in these leafy green vegetables, including zinc (Zn), copper (Cu), and manganese (Mn) (Figure 5b).

## 4 DISCUSSION

In this study, multiple leafy green vegetables, including lettuce, and Swiss chard, showed significant levels of arsenic uptake when grown directly in garden soil, while other plant samples such as tomato and Brussel sprouts showed no or below threshold heavy metal and arsenic levels. Previous studies have shown that leafy green vegetables are more prone to heavy metal and metalloid contamination than many other vegetables (Beavington, 1975; Sharma, Katnoria, & Nagpal, 2016; Sterrett et al., 1996). The increased heavy metal content of vegetables with large leaf surfaces may be partially attributed to the increased surface area for heavy metal containing dust particles to settle on the leaf, although this may also increase internal metal content through foliar transfer (Chaney, Sterrett, & Mielke, 1984; Harrison & Chirgawi, 1989; Schreck et al., 2012). However, studies performed in glasshouses with filtered air show an enhanced uptake of heavy metals in these plants (Li, Shi, Jin, Wu, & Sheng, 2017; Preer et al., 1980). Additionally, rinsing plant tissues with distilled water, as conducted in this study, before heavy metal measurements has been shown to reduce the presence of this surface dust contamination (Norouzi, Khademi, Faz Cano, & Acosta, 2015). Fruit trees grown in contaminated soils will commonly take up heavy metals and metalloids from the environment into the plant tissue (Madejón, Marañón, & Murillo, 2006; Peryea, 2001). Uptake of heavy metals and metalloids occurs due to chemical similarities to essential nutrients resulting in unintended uptake through root transporters (Korshunova, Eide, Clark, Guerinot, & Pakrasi, 1999; Wu, Ren, McGrath, Wu, & Zhao, 2011). The amount of heavy metal and metalloid uptake can vary greatly depending on the type of tree (Pulford & Watson, 2003). However, for the tree species analyzed, we found that the edible fruit tissue did not accumulate heavy metals and arsenic compared with the leaf tissues under our conditions (Figures 3, 4, 5), which is consistent with other studies (Ademoroti, 1986; Li et al., 2006).

In the leaf tissues collected from the Black Mission fig and Mexican lime trees, lead levels between 1 and 1.5 mg/kg dry weight were detected. Measurements of dry weight of samples showed that this was equivalent to approximately 0.2 to 0.3 mg/kg fresh weight in Black Mission fig leaves and 0.3 to 0.5 mg/kg fresh weight in lime leaves. While the United States Food and Drug Administration (FDA) has no limits or recommendations for lead contamination in fruits or vegetables, there are limits for drinking water (5 µg/kg lead) and candy intended for children (100 µg/kg lead). The Black Mission fig and Mexican lime sample fresh weight concentration exceeded the limit for drinking water by approximately 40‐ to 100‐fold.

For the leafy green vegetables grown directly in the ground, arsenic levels of 0.42 ± 0.055 mg/kg dry weight (≈0.084 mg/kg fresh weight) for lettuce and 0.80 + 0.073 mg/kg dry weight (≈0.15 mg/kg fresh weight) for Swiss chard were detected. As with lead, there are no FDA fruit or vegetable standards for arsenic. However, there are limits for drinking water (10 µg/kg) and recommendation levels for infant rice cereals (100 µg/kg). The United Nations Food and Agriculture Organization has recommended a limit of 0.2 mg/kg inorganic arsenic in polished rice (Commission, 2014). The detected fresh weight levels were up to 50% higher than recommended arsenic levels in rice cereals and exceeded drinking water limits approximately eightfold to 15‐fold. In 2012, Consumer Reports conducted a study to investigate arsenic levels in over 200 samples of commercial rice products and found that a single serving of some rice products contained arsenic levels that would match exposure to a full day of drinking water with 5 ppb arsenic, the EPA recommended exposure limit (Arsenic in Your Food, 2012).

There are multiple factors that have led to the limited safety regulations surrounding heavy metal and metalloid contamination in foods. First, there are no medical or research data showing that there are safe levels of lead for human consumption. Second, while there are recommendations for total weekly lead intake based on body weight, the safe limits for each individual item would vary depending on each individual's diet and weight, as well as the proportion of an individual's diet each item represents. Finally, safe limits set for lead and arsenic levels in food and water are often based on what is feasible to achieve rather than what is ideal for human health, leading to less regulation than might otherwise be expected.

In several of the leaf samples taken from the fruit trees, there were detectable levels of chromium, as high as 1.25 mg/kg. However, chromium presents unique difficulties in assessing risk due to its existence in two different forms, Cr (III) and Cr (VI) with vastly different toxicities (Gad, 1989). Hexavalent chromium (Cr [VI]) is substantially more toxic to human health than Cr (III) due to its greater ability to be transported into cells (Wilbur et al., 2012). The extraction methods used in this study do not allow for the distinction between Cr (III) and Cr (VI) in the plant tissues, which limits our ability to make determinations about the actual risks associated with the detected levels.

### 4.1 Recommendations for community gardens

Based on the results found in this study, as well as other urban garden and brownfield studies, we recommend testing of toxic heavy metals and arsenic levels in edible plant samples. It is clear that the specific combination of plant species grown and growth methods should be customized based on potential or known contamination risks. High metal accumulating vegetables such as leafy green vegetables should be grown in uncontaminated soils, which in an urban garden setting will often require the construction of raised beds, the purchase and use of clean soil, removal of the contaminated top soil, and addition of a barrier that shields the new soil from the contaminated soil. However, there are some limitations to the use of raised beds; raised beds do not remove existing contamination, making it a temporary rather than long‐term solution. Continued testing for contamination is recommended, to monitor for breakdown of barriers between raised bed and ground soil, if present. Planting of crops with long roots can also lead to penetration into contaminated soils. Also, warning signs indicating the soil contamination should be posted on sites utilizing this method. While fruit trees in the present study appeared to minimize the transfer of heavy metals and arsenic into the edible fruit tissues, variation in the soil concentration and specific soil contamination, tree species, and tree age indicates that there are risks of contamination that need to be addressed by testing of fruit samples.

For groups interested in testing for contaminants present in soil, there are a number of companies that can be contracted to perform measurements; however, the costs of professional testing can be prohibitive. As an alternative, community events, such as Soil Kitchen, that do less thorough soil testing have been conducted in neighborhoods. At these events, residents are able to bring in soil from their community or backyard garden for testing and are often given a free meal while they wait for results. Testing of heavy metals and metalloids in contaminated edible plant tissue remains a resource intensive process. The main limiting factor for soil and plant testing is access to resources, including equipment, such as inductively coupled spectrometers and X‐ray fluorescence analyzers, as well as the training to conduct measurements and interpret results. One avenue to overcome these limitations is to develop a partnership between local universities and community organizers that allows local residents to tap into the wealth of resources available, while also giving university researchers an avenue to apply their work in the community.

## 5 CONCLUSIONS

In the present study, we investigate the content of toxic heavy metals and the metalloid arsenic in several crops grown in an urban community garden. We found detectable levels of lead in leaf tissues (0.2–0.5 mg/kg dry weight) of some fruit trees grown on the site. Crops grown directly in the ground had detectable arsenic, ~0.4 mg/kg dry weight in lettuce and ~0.8 mg/kg dry weight in Swiss chard; however, crops grown in raised soil beds had no detectable arsenic contamination. While converting urban brownfields into community gardens and urban green spaces does pose potential risks related to contaminant exposure, including exposure to heavy metals and metalloids, the potential benefits to the community should not be ignored (Kingsley, Townsend, & Henderson‐Wilson, 2009; Litt et al., 2011). Community gardens especially in food desert areas can provide an essential source of healthy fruits and vegetables needed to maintain a healthy diet. However, testing of soils and food samples is highly recommended. In addition to minimizing health risks associated with proper diet, community gardens also provide a space to build positive social connections in the community (Twiss et al., 2003). By combining a place‐based approach to healthy‐eating strategies with community interaction and engagement, the larger community health can be enhanced, fostering cultural exchange and a more holistic understanding of the interactions between food and health (Armstrong, 2000; Draper & Freedman, 2010).

## CONFLICT OF INTEREST

The authors declare no conflict of interest associated with the work described in this manuscript.

## AUTHOR CONTRIBUTIONS

AC, PW, KP, and JS designed the research. AC, DF, FA, QY, DZ, AJS, and YZ performed the research. IZ and AW contributed analytical and computational tools. AC, DF, FA, QY, and AJS analyzed data. AC, KP, QY, and JS wrote the paper.
