# Supplementary material for: Monitoring and mitigation of toxic heavy metals and arsenic accumulation in food crops: A case study of an urban community garden
Source: Plant Direct. 2020 Jan 14;4(1):e00198. doi: 10.1002/pld3.198 (PMC6957986; doi:10.1002/pld3.198)
Supplement: Supplementary file 1 [file PLD3-4-e00198-s001.pdf]

WALLACE LABORATORIES, LLC
365 Coral Circle
El Segundo, CA 90245
phone (310) 615-0116 fax (310) 640-6863

June 26, 2013

Keith Pezzoli, kpezzoli@ucsd.edu
UCSD
9500 Gilman Drive
M/C 0521
La Jolla, CA 92093

RE: 4540 Ocean View Blvd, San Diego

Dear Keith,

The average pH is near neutral at 6.87. The pH values range from 6.02 to 7.77. Samples 7 and 8 have the highest alkalinity with pH values of 7.73 and 7.77, respectively. The others range form moderately acidic to slightly alkaline.

Salinity is moderate at 1.36 millimho/cm on average. Salinity ranges from 0.19 to 4.79 millimho/cm. Samples 9 and 10 have the highest salinity at 4.79 and 4.51 millimho/cm, respectively. Chloride is high at 516 and 502 parts per million in the saturation extract. Salt-sensitive plants need chloride below about 150 parts per million. Chloride can be lowered with leaching of the soil. Chloride is 369 parts per million in the saturation extract of sample 3.

The average SAR (sodium adsorption ratio) is 3.5. SAR ranges from 0.6 to 6.1. Samples 3, 5, 6, 7 and 9 have SAR over 4.5. High SAR values have adverse effects on soil physical properties including reduced water percolation, decreased soil aggregate stability, increased clay dispersion, increased swelling of expandable clays, increased surface crusting and reduced soil tilth. High sodium also restricts the uptake of competitive ions such as potassium. SAR can be lowered with the addition of gypsum and with leaching.

The Surface Composite sample has high fertility except for low mineral nitrogen. The pH is 6.92. Salinity is 0.80 millimho/cm. SAR is 1.3. Sulfur is modest.

Samples 1) Back West Corner and 9) 3E also have moderate phosphorus and potassium. The other samples have low or modest phosphorus and potassium. Nitrogen is high in sample 9 and is moderate in sample 3. Iron is low in 5 samples. Zinc is slightly low in sample 10. Manganese is low in sample 7. Boron is modestly low on average. Micronutrients can be applied with the addition of a humus type of compost. Gypsum is present in samples 9 and 10.

A modest level of lead is present in sample 1. It is not expected to restrict growth.

Recommendations

Clean up the soils as needed. Remove any existing vegetation that will not remain and major roots larger than about 1 inch in diameter. Buried vegetation and buried roots readily decompose and frequently become putrid. Protect the existing roots of trees which are to remain in the current planting areas. Remove any turf and thatch layer of the turf. If present. Remove any pockets of gravel, rocks, asphalt, debris, etc.

General soil preparation for turf, ground cover and shrubs

Cultivate the soil at least 12 inches deep on 12 inch centers. Reduce soil clods to a maximum diameter of 1 inch in the top 6 inches. Do not till muddy soils, they are not friable. Optimum moisture content is partially damp. The moisture content should not be so great that excessive compaction will occur, nor so dry that clods will not break readily. Remove rocks, gravel, debris and clods larger than 1 inch in diameter from the top 6 inches. Lower the gravel content to a maximum of 20%.

Application of amendments and fertilizers.

Uniformly broadcast the following materials.
The rates are per 1,000 square feet:

Potassium sulfate (0-0-50) – 6 pounds except 1, 9 and 11
Triple superphosphate (0-45-0) – 4 pounds except 1, 9 and 11
Gypsum – 30 pounds except 9 and 10
Organic amendment – about 3 cubic yards, sufficient amount to provide soil organic matter in the range of 4% to 7% on a dry weight basis

Homogeneously incorporate the above materials into the soil to a depth of six inches. The soil organic matter needs to be stable in order to avoid excessive decomposition. Fine rake the soils after soil preparation and remove gravel larger than 3/8 inches in diameter from the top several inches.

After the preparation of the soil, the quality of the amended soil can be tested for suitability prior to seeding and planting.

Preparation of soil for backfilling trees and container plants

Prepare planting pits normally twice as wide as the rootballs. The walls and bottom of the planting pits should not have compacted soil except under the rootball. If necessary, loosen glazed soil by scarifying the soil surface.

Blend the following materials into clean excavated soil. Remove rocks, gravel, debris and clods larger than 1 inch in diameter. Excessive gravel should not be present. The general maximum is 20%. Rates are per cubic yard:

Potassium sulfate (0-0-50) – 1/4 pound except 1, 9 and 11
Triple superphosphate (0-45-0) – 1/4 pound except 1, 9 and 11
Gypsum – 1.5 pounds except 9 and 10
Organic amendment – about 15% by volume, sufficient amount to provide soil organic matter in the range of 4% to 7% on a dry weight basis

Backfill the transplant with the prepared soil.

Organic amendment suggestions

1. Humus material shall have an ash content of no less than 6% and no more than 20%. Organic matter shall be at least 50% on a dry weight basis.
2. The pH of the material shall be between 6 and 7.5.
3. The salt content shall be less than 10 millimho/cm @ 25° C. (ECe less than 10) in a saturated paste extract.
4. Boron content of the saturated extract shall be less than 1.0 parts per million.
5. Silicon content (acid-insoluble ash) shall be less than 50%.
6. Calcium carbonate shall not be present if to be applied on alkaline soils.
7. Types of acceptable products are composts, manures, mushroom composts, straw, alfalfa, peat mosses etc. low in salts, low in heavy metals, free from weed seeds, free of pathogens and other deleterious materials.
8. Composted wood products are conditionally acceptable [stable humus must be present]. Wood based products are not acceptable which are based on red wood or cedar.
9. Sludge-based materials are not acceptable.
10. Carbon:nitrogen ratio is less than 25:1.
11. The compost shall be aerobic without malodorous presence of decomposition products.
12. The maximum particle size shall be 0.5 inch, 80% or more shall pass a No. 4 screen.

Maximum total permissible pollutant concentrations in amendment in parts per million on a dry weight basis:

arsenic	20	copper	150	selenium	30
cadmium	15	lead	100	silver	10
chromium	100	mercury	10	vanadium	200
cobalt	50	molybdenum	20	zinc	200
		nickel	100		

Leach the soils with high salinity, chloride, and sodium. Lower the salinity to less than 3 millimho/cm. Lower the chloride to less than 150 parts per million in the saturation extract for salt-sensitive plants. Lower the SAR to less than 3. Afterwards apply nitrogen as listed below for maintenance fertilization.

For site maintenance fertilization, apply ammonium sulfate (21-0-0) at 5 pounds per 1,000 square feet quarter in alkaline areas or Yara or Simplot calcium ammonium nitrate (27-0-0) at 4 pounds per 1,000 square feet for pH neutral areas. Adjust the maintenance program as needed.

Sincerely,

Garn A. Wallace, Ph. D.
GAW:n
