# Supplementary material for: Monitoring and mitigation of toxic heavy metals and arsenic accumulation in food crops: A case study of an urban community garden
Source: Plant Direct. 2020 Jan 14;4(1):e00198. doi: 10.1002/pld3.198 (PMC6957986; doi:10.1002/pld3.198)
Supplement: Supplementary file 2 [file PLD3-4-e00198-s002.pdf]

Reviewer #1:

Risks associated with urban gardening are not receiving enough attention yet, so it is good that possible contamination by toxic metals and metalloids is addressed in this study. Deriving recommendations for community gardens is particularly important.
I have a couple of points that in my view require attention:
1. Please report limits of detection for the ICP-OES analysis (which is not that sensitive).

Thank you for this comment. We have now added detection limit information in the Methods section.

2. "This increased uptake of heavy metals and metalloids in leafy green vegetables is believed to be associated with their overall higher metal content when compared to other fruits and vegetables."
I am not aware that overall the metal content is that much higher. Zn concentrations, for example, do not vary that much between plants and plant organs because of tight control. Can the authors cite evidence? The data reported here do not support this either (with the exception of swiss chard). An alternative explanation is the larger surface area and aerial deposition. Generally, the detection of Pb is often explained by dust particles etc. deposited on the leaves. This should be discussed.

The reviewer raises relevant points. We have deleted the above sentence. In addition, in the first paragraph of the Discussion we have added text referring to washing of plant tissue surfaces prior to measurements, greenhouse studies and also foliar absorbance of metals. In addition, we have added information in the Methods that tissue samples were washed with double-distilled water.

3. The following cannot be generally stated as it depends on individual diets. Also, I did not understand why a number is given: "In areas with no contamination of drinking water, ingestion is the highest risk of arsenic exposure (0.38 µg/(kg day)), with vegetables making up the largest percentage of exposure, followed by fruit & fruit juices, and rice (Xue et al. 2010; Chain 2009)."

To address this helpful comment we have removed the number and have reworded the sentence to make more clear that we are referring to reported conclusions from the cited publications.

4. The discussion would benefit from including more numbers on contamination levels found in other studies such as market surveys.

We have added reference to a study published in Consumer Reports, in which arsenic levels were measured in over 200 samples of commercial rice products. While our manuscript is not a review of previous studies, we cite related research throughout the

manuscript and are happy to include any studies. The Consumers Report study we have added was quite extensive and widely used.

Reviewer #2:

This study investigated the uptake of various heavy metals by fruits and vegetables grown in an urban community garden. Although the issue related to the health risk of heavy metals in the soils of urban community gardens is important, the uptakes of heavy metals by crop plants, however, have been extensively studied, and abundant information can be found in the literature. Thus, if the results only showed the heavy metals contents of fruits and vegetables grown in a specific soil contaminated by heavy metals, the novelty of this study seems to be not enough.

We appreciate this comment. We note that *Plant Direct* has been designed to publish data and studies even if other studies have appeared on the subject, including publishing papers in disciplines not covered by the parent journals of ASPB and SEB. Furthermore, the present study is specifically relevant to the many and growing numbers of urban food growers and we will work to ensure that this study is widely disseminated, which can add to the growing impact of *Plant Direct*. We thank the reviewer for their feedback on this subject

Unlike farmland soils, which are used to grow only one or two types of crops continuously, the variety of crop plants grown in urban community gardens seems to vary from time to time. I am wondering how the soils in urban community gardens are different from farmland soils, in terms of soil depth (and its effect on root development and metal uptake) and the management of crop residues, soil and irrigation water (and their effects on the bioavailability of heavy metals).

Soils can be very diverse in chemical nature. Nevertheless nutrients, cadmium and arsenic are absorbed into plant tissues under many naturally occurring conditions. To address the reviewer’s comment, we have added information on the history of this site, which was used by the adjacent (directly neighboring) company that may have contributed to contamination of the soil. We have further added the original report as Supplemental file on analyses conducted by the cited company which includes more information on the soil samples analyzed at the site, prior to the begin of the present study of gardened food crops. We now further refer to soil pH measurements and other parameters from this report in the revised manuscript.

In the experimental section, some details are missing. The preparation and treatments of soil for the analysis of heavy metal content were not described. The procedures of quality control and quality assurance need to be provided. Meanwhile, "detectable" and "nondetectable" mentioned in the results and discussion should be based on the detection limit of the method of heavy metal analysis.

The Methods section of the manuscript describes the procedures used for measuring the soil samples. We have further now added the original report on analyses conducted by the cited company, which includes more information on the soil samples analyzed at the site, prior to the begin of the present study of gardened food crops. We have also added information on the detection limits to the Methods section.

I will suggest the authors analyze the basic properties of the soils (such as pH, organic matter content, CEC, etc.), because these properties determine the bioavailability of heavy metals in soils.

The reviewer’s comment is appreciated. We have further added the original report on analyses conducted by the cited company, which includes information on the soil samples analyzed at the site. We note that the present study will be useful to urban community gardeners, who may have divergent soils. The relevant focus of our study is to report on heavy metal, arsenic and nutrient metal levels in the edible plant tissues. This study will further be helpful for urban gardeners, who are interested in elements that may be appearing in their foods and how to address this important issue.

Statistical analysis needs to be conducted to see whether the results are statistically significant or not. Meanwhile, when mean and standard deviation are reported, the significant digits of both values have to be consistent.

Statistical analyses are now included. ANOVA analyses were conducted as described in the figure legends. Figure 2 shows soil sampling performed by the reported company prior to the beginning of this study. Single soil samples were tested, as described in the manuscript and the Supplemental file report form the company. Since these were ten separate single soil samples, no statistical data are included for these data. If preferred this figure could be moved to the Supplemental materials. The focus of the present study is the measurement of the provided nutrient and toxic elements in the described plant species and tissues at this site and shows mitigation methods and provides advice to community gardeners. As natural soils can be variable, but plants can accumulate nutrients and toxicants quite effectively, we feel that the soil provide additional information together with the Supplemental company information, for those readers who are interested.

Although arsenic is a metalloid, "heavy metals and arsenic" can be simplified to be "heavy metals."

We thank the reviewer for this comment. As arsenic is a metalloid, we prefer this terminology.

--------------------------------------------------------------------------- Editor comments:
Both reviewers request to describe some technical details. Comparison between crops produced in the farm and community gardens with the information of soil property and published data in other studies is highly suggested.

Thank you for the feedback.We have added data and information and cited research as described in our responses to the reviewer comments and have added a Supplemental file describing the soil properties. We would be happy to add citation of additional relevant studies proposed by the editor.
